# Efficient inference for sparse latent variable models of transcriptional regulation

**DOI:** 10.1093/bioinformatics/btx508

**Published:** 2017-08-26

**Authors:** Zhenwen Dai, Mudassar Iqbal, Neil D Lawrence, Magnus Rattray

**Affiliations:** 1 Department of Computer Science, University of Sheffield, Sheffield, UK; 2 Amazon Research, Cambridge, UK; 3 Division of Informatics, Imaging & Data Sciences, Faculty of Biology, Medicine, and Health Sciences, University of Manchester, Manchester, UK

## Abstract

**Motivation:**

Regulation of gene expression in prokaryotes involves complex co-regulatory mechanisms involving large numbers of transcriptional regulatory proteins and their target genes. Uncovering these genome-scale interactions constitutes a major bottleneck in systems biology. Sparse latent factor models, assuming activity of transcription factors (TFs) as unobserved, provide a biologically interpretable modelling framework, integrating gene expression and genome-wide binding data, but at the same time pose a hard computational inference problem. Existing probabilistic inference methods for such models rely on subjective filtering and suffer from scalability issues, thus are not well-suited for realistic genome-scale applications.

**Results:**

We present a fast Bayesian sparse factor model, which takes input gene expression and binding sites data, either from ChIP-seq experiments or motif predictions, and outputs active TF-gene links as well as latent TF activities. Our method employs an efficient variational Bayes scheme for model inference enabling its application to large datasets which was not feasible with existing MCMC-based inference methods for such models. We validate our method on synthetic data against a similar model in the literature, employing MCMC for inference, and obtain comparable results with a small fraction of the computational time. We also apply our method to large-scale data from *Mycobacterium tuberculosis* involving ChIP-seq data on 113 TFs and matched gene expression data for 3863 putative target genes. We evaluate our predictions using an independent transcriptomics experiment involving over-expression of TFs.

**Availability and implementation:**

An easy-to-use Jupyter notebook demo of our method with data is available at https://github.com/zhenwendai/SITAR.

**Supplementary information:**

Supplementary data are available at *Bioinformatics* online.

## 1 Introduction

A typical biological study of cellular response to external stress/stimuli or certain knock-outs leads to the measurement of gene expression patterns of thousands of differentially expressed genes (Galagan *et al.*, 2013; Nieselt *et al.*, 2010). Furthermore, transcription factor binding sites data from literature as well as *de novo* computational motif predictions (Gama-Castro *et al.*, 2016; Sierro *et al.*, 2008; Studholme *et al.*, 2004), in the case of small prokaryotic genomes, are accessible for many well-studied organisms. Large-scale ChIP-seq assays, e.g. (Galagan *et al.*, 2013; Minch *et al.*, 2015) are also available, detailing the genome-wide binding patterns of specific transcription factor proteins (TFs). A subsequent computational and statistical challenge is to integrate these data in order to obtain a quantitative picture of the underlying regulatory interactions between TF proteins and target genes. In the last decade, many statistical methods have been proposed (see Marbach *et al.*, 2010 for a review) which infer gene regulatory networks by exploiting correlation patterns in the gene expression data. However, mRNA expression data alone cannot disentangle the complex wiring of regulatory interactions (Marbach *et al.*, 2012). Other experimental techniques for elucidation of regulatory interactions also have limitations, e.g. ChIP-seq experiments do not determine the effect of TF binding events on target genes and it is difficult to distinguish direct versus indirect regulatory effects in TF perturbation experiments (Siahpirani and Roy, 2017). It is therefore necessary to integrate different genomic datasets in order to infer context-specific regulatory networks.

TF proteins may be regulated at the post-transcriptional level and therefore an important consideration in modelling transcriptional regulation is that measured RNA levels often do not provide a good proxy for the concentration of active TFs. Bayesian statistical methods, especially sparse latent factor models (Carvalho *et al.*, 2008; Iqbal *et al.*, 2012; Pournara and Wernisch, 2007; Sabatti and James, 2006; Sanguinetti *et al.*, 2006) which are the main focus of this study, offer a flexible framework for data integration. These methods treat the regulator activities as latent (unobserved) variables which can be inferred from the RNA expression levels of their target genes. Sparse latent factor models also have other biological applications, e.g. modelling cellular heterogeneity in single-cell RNA-seq data (Buettner *et al.*, 2015). The core underlying hypothesis in the context of transcriptional regulation is that a large number of observed gene expression profiles can be explained by the unobserved activities of a small number of regulatory proteins. Biologically meaningful prior information on the underlying transcriptional regulatory network (between TFs and genes) can be obtained from computational motif predictions (Li *et al.*, 2002; Studholme *et al.*, 2004) or large-scale ChIP-seq experiments (Galagan *et al.*, 2013). Sparse factor models combine the prior network with relevant gene expression data in order to infer the true underlying regulatory connections driving gene expression in the experiment under study, as well as the activities of the regulators and the strength of regulatory effects.

Despite the appeal of sparse factor models for biological applications, inference in these models presents a computational challenge. Markov Chain Monte Carlo (MCMC) can be used to carry out model inference (Iqbal *et al.*, 2012; Sabatti and James, 2006) and has the advantage of quantifying uncertainty in all the inferred parameters. However, MCMC suffers from convergence issues, and becomes computationally prohibitive even for a moderate number of regulators. This lack of scalability hinders the application of sparse factor models, since a typical biological experiment involves many dozens of TFs and thousands of genes. As more ChIP-seq and gene expression data becomes available, efficient methods are therefore needed to extract biological information from these data.

Here, we present a novel method in the family of sparse factor models, named SITAR (Sparse latent varIable model of TrAnscritional Regulation), in which a spike-and-slab prior is used to induce sparsity in network connections. We propose an efficient variational inference method by deriving a closed-form variational lower-bound for our model. This adaptation of the inference scheme enables us to scale up the inference over much larger datasets than current methods based on MCMC can cope with. We test our method on synthetic data against a similar published method which uses MCMC-based inference. We then apply our method to a large-scale dataset (Galagan *et al.*, 2013; Minch *et al.*, 2015) from *Mycobacterium tuberculosis* (MTB) with ChIP-seq data for 113 TFs and matched gene expression data for 3863 genes, which include multiple time series covering hypoxia and over-expression experiments for some TFs. This is one of the largest application of its kind and the running time for our method for this dataset was about 7 h on a laptop.

The paper is organized as follows. In Section 2, we describe our model for integrating binding sites and gene expression data. We describe the choices of the prior on model parameters and present the variational inference algorithm and method for recovery of latent activities. In Section 3 we describe validation results on synthetic data and results on an application to a large-scale real dataset from MTB. We report biological validation of our predictions on the MTB dataset by comparing our inference results to results from an independent TF over-expression study which was not used for learning the model.

## 2 Materials and methods

We model gene expression as a weighted sum of TF activities: $e_{it}=\sum_{j=1}^{L}a_{ij}p_{jt}+\epsilon_{it}$, where *e_it_* represents the expression of gene *i* in experiment *t*, *a_ij_* is the control strength of TF *j* on gene *i*, *p_jt_* is a proxy for the concentration of active form of TF *j* in experiment *t* and ϵ_*it*_ accounts for measurement errors and biological variation. In matrix notation the model is formulated as

(1) E=AP+ϵ,

where $E\in R^{N\times M},\;A\in R^{N\times L},\;P\in R^{L\times M}$, *N* is the number of genes, *M* is the number of experiments and *L* is the number of TFs. Both the control strength of TFs, A, and the concentration of active TFs, P, are unknown. By assuming that the noise ϵ follows an *i.i.d.* Gaussian distribution, we can define the distribution of the expression data E as

(2) $$p(E|A,P)=\prod_{t=1}^{M}N\left(E_{t}|AP_{t},\sigma^{2}I\right),$$

where $E_{t}$ and $P_{t}$ indicates the *t*th column of E and P, and $\sigma^{2}$ is the variance of Gaussian noise.

We define a unit variance Gaussian prior on the elements of P, i.e. $p(P_{t})=N\left(P_{t}|0,I\right)$, and marginalize out P from Equation (2):

(3) $$p(E|A)=\prod_{t=1}^{M}N\left(E_{t}|0,AA^{⊤}+\sigma^{2}I\right).$$

Only a small subset of genes are controlled by individual TFs due to biological constraints and therefore A is known to be sparse. To keep inference tractable we introduce some hard constraints on the allowed connections through a binary connectivity matrix $X\in R^{N\times L}$ which is obtained from motif analysis or ChIP-seq data (as explained in Section 3). Entry $x_{ij}=0$ indicates that TF *j* cannot control gene *i*, i.e. $a_{ij}=0$. However, even if a connection is allowed by the connectivity matrix it may not be active, e.g. when $x_{ij}=1$ then TF *j* does not necessarily control the corresponding gene *i*. To model this we introduce a latent binary variable for each pair of TF and gene, $S\in R^{N\times L}$, which control the connections between TFs and genes. The probability distribution of the expression matrix is modified to be:

(4) $$p(E|A,S)=\prod_{t=1}^{M}N\left(E_{t}|0,(A{}^{\circ}S){\left(A{}^{\circ}S\right)}^{⊤}+\sigma^{2}I\right),$$

where A°S indicates the element-wise multiplication between A and S. We incorporate the information of the connectivity matrix into the prior distribution of these binary variables. For the entries of *S* with $x_{ij}=0$, we set $p(s_{ij})=1-s_{ij}$. For $x_{ij}=1$, we assume $s_{ij}$ has a prior probability π_*j*_, $p(s_{ij}|\pi_{j})=\pi_{j}^{s_{ij}}{\left(1-\pi_{j}\right)}^{1-s_{ij}}$, and π_*j*_ follows a beta prior $p(\pi_{j})=\text{Beta}(2,2)$. Finally, with a unit Gaussian prior distribution for $A,\;p(a_{ij})=N\left(a_{ij}|0,1\right)$, the marginal likelihood distribution for our model is

(5) p(E)=∫p(E|A,S)p(A)p(S|π)p(π)dAdSdπ.

Given expression data E, we can then write the posterior distribution of the regulatory interactions using Bayes rule:

(6) $$p(A,S|E)=\frac{\int p(E|A,S)p(A)p(S|\pi )p(\pi )d\pi }{p(E)}.$$

From this, we can estimate A and S by computing their expections ${\langle A\rangle }_{p(A,S|E)}$ and ${\langle S\rangle }_{p(A,S|E)}$, and the posterior also provides credible regions for these estimates.

### 2.1 Variational inference

As mentioned above, our aim is to infer the posterior distribution of the regulatory network S, the control strength A and the activity profiles of transcription factors P by observing gene expression data E. Unfortunately, exact inference of the posterior is infeasible due to the intractable integral in Equation (5). Sampling-based approaches such as Markov Chain Monte Carlo (MCMC) have been developed (Iqbal *et al.*, 2012) but are very time-consuming and prohibitively slow for large-scale datasets, e.g. thousands of genes and hundreds of TFs. In this work, we propose an efficient inference algorithm based on a variational approximation which reduces the computational run-time for large datasets from weeks to hours.

Variational inference avoids the evaluation of the intractable marginal likelihood by optimizing parametric posterior distributions with respect to a lower bound of the log marginal likelihood. We assume a variational posterior distribution q(A,S,π) and derive a lower bound such as

(7) $$\text{log}\;p(E)\geq \int q(A,S,\pi )\;\text{log}\;\frac{p(E|A,S)p(A)p(S|\pi )p(\pi )}{q(A,S,\pi )}dAdSd\pi .$$

For our model, the standard mean-field approximation q(A,S,π)=q(A)q(S)q(π) is still intractable due to the covariance matrix inversion in Equation (4). In this paper, we exploit the fact that our model can be viewed as a Gaussian Process latent variable model (Lawrence, 2005) with a linear kernel and a spike-and-slab prior. This enables us to adopt the sparse Gaussian Process formulation (Titsias, 2009; Titsias and Lawrence, 2010) for our model. We first rewrite our likelihood expression (4) in the form of a Gaussian Process (GP):

(8) $$p(E|F)=\prod_{t=1}^{M}N\left(e_{t}|f_{t},\sigma^{2}I\right),$$

(9) $$p(f_{t}|A,S)=N\left(f_{t}|0,K_{ff}\right),$$

where F is the noise-free observation of the gene expression data E and $K_{ff}$ is the covariance matrix of F computed according to our model, i.e. $K_{ff}=(A{}^{\circ}S){\left(A{}^{\circ}S\right)}^{⊤}$. The sparse GP approximation introduces an auxiliary latent variable $U\in R^{L\times L}$ with a corresponding inducing input $I\in R^{L\times L}$ (I is an identity matrix.) This allows us to reformulate the prior distribution of F in terms of the auxiliary variable:

(10) $$p(f_{t}|u_{t},A,S)=N\left(f_{t}|K_{fu}K_{uu}^{-1}u_{t},K_{ff}-K_{fu}K_{uu}^{-1}K_{fu}^{⊤}\right),$$

(11) $$p(u_{t})=N\left(u_{t}|0,K_{uu}\right),$$

where the conditional distribution (10) is derived through GP inference and $K_{uu}$ and $K_{fu}$ are the covariance matrices, i.e. $K_{uu}=XX^{⊤}$ and $K_{fu}=(A{}^{\circ}S)X^{⊤}$. Note that marginalizing out the auxiliary variable U in Equations (10) and (11) returns the original distribution of F in Equation (9). Following the sparse GP formulation, we define the variational posterior distribution as q(F,U,A,S,π)=p(F|U,A,S)q(U)q(A,S)q(π) and obtain a lower bound of the marginal likelihood:

(12) $$\begin{array}{c} L=F-\text{KL}\left(q(U)\;||\;p(U)\right)-\text{KL}\left(q(A,S)q(\pi )\;||\;p(A)p(S|\pi )p(\pi )\right), \end{array}$$

where $F={\langle \;\text{log}\;p(E|F,U,A,S)\rangle }_{p(F|U,A,S)q(A,S)q(U)}$. Since A and S are often strongly correlated in the posterior distribution, their variational posterior is defined as a conditional distribution,

$$q(S)=\prod_{i=1}^{N}\prod_{j=1}^{L}\gamma_{ij}^{s_{ij}}{\left(1-\gamma_{ij}\right)}^{\left(1-s_{ij}\right)},$$

(13) $$q(a_{ij}|s_{ij}=1)=N\left(a_{ij}|\mu_{ij},c_{ij}\right),$$

where γ_*ij*_ is the posterior probability of TF *j* controlling the gene *i* and μ_*ij*_ and *c_ij_* are the posterior mean and variance of the control strength. Note that the distribution $q(a_{ij}|s_{ij}=0)$ is not defined explicitly, because, as the switch variable is zero, the control strength does not influence the likelihood anymore, so that $q(a_{ij}|s_{ij}=0)$ will only appear inside the KL divergence, which makes it always equal to the prior distribution p(A). With the above posterior distribution of q(A,S), the first expectation in Equation (12) can be solved analytically.

(14) $$\begin{array}{r} {\langle \;\text{log}\;p(E|F,U,A,S)\rangle }_{p(F|U,A,S)q(U)q(A,S)}=-\frac{NM}{2}\text{log}\;2{\pi \sigma }^{2}\\ -\frac{1}{2\sigma^{2}}{\langle \sum_{t=1}^{M}u_{t}^{⊤}K_{uu}^{-1}\Psi_{2}K_{uu}^{-1}u_{t}\rangle }_{q(U)}\\ +\sum_{t=1}^{M}\frac{1}{\sigma^{2}}e_{t}^{T}\Psi_{1}K_{uu}^{-1}{\langle u_{t}\rangle }_{q(U)}-\frac{1}{2\sigma^{2}}\sum_{t=1}^{M}e_{t}^{⊤}e_{t}\\ -\frac{M}{2\sigma^{2}}\psi_{0}+\frac{M}{2\sigma^{2}}\text{Tr}(K_{uu}^{-1}\Psi_{2}) \end{array}$$

where ψ_0_, $\Psi_{1}$ and $\Psi_{2}$ denote the expectation of the covariance matrices w.r.t. q(A,S), i.e. $\psi_{0}=\text{Tr}({\langle K_{ff}\rangle }_{q(A,S)}),\;\Psi_{1}={\langle K_{fu}\rangle }_{q(A,S)},\;\Psi_{2}={\langle K_{fu}^{⊤}K_{fu}\rangle }_{q(A,S)}$. For the linear kernel used in this paper, ψ_0_, $\Psi_{1}$ and $\Psi_{2}$ can be derived analytically (we call them psi-statistics) as:

(15) $$\psi_{0}=\sum_{i=1}^{N}\sum_{j=1}^{L}\gamma_{ij}(\mu_{ij}^{2}+c_{ij}),$$

(16) $${\left(\Psi_{1}\right)}_{id}=\sum_{j=1}^{L}\gamma_{ij}x_{dj}\mu_{ij},$$

(17) $$\begin{array}{l} {\left(\Psi_{2}\right)}_{dd\prime }=\sum_{i=1}^{N}(\sum_{j=1}^{L}{\text{γ}}_{ij}z_{dj}z_{d\prime j}({\text{μ}}_{ij}^{2}+c_{ij})\\ +\sum_{j=1}^{L}\sum_{j\prime \neq j}{\text{γ}}_{ij}{\text{γ}}_{ij\prime }z_{dj}z_{d\prime j\prime }{\text{μ}}_{ij}{\text{μ}}_{ij\prime }). \end{array}$$

The optimal distribution of q(U) can be derived analytically from Equation (12) by setting its derivative to be zero:

(18) $$q(u_{t})=N\left(u_{t}|K_{uu}{\left(K_{uu}+\Psi_{2}\right)}^{-1}\Psi_{1}^{T}e_{t},K_{uu}{\left(K_{uu}+\Psi_{2}\right)}^{-1}K_{uu}\right).$$

By substituting the optimal variational distribution of q(U), the variational lower bound can be formulated in closed form which enables us to perform inference efficiently by optimizing the model parameters and variational parameters with respect to the closed-form lower bound.

### 2.2 Recovering the activities of TFs

Using the lower bound of the log-marginal likelihood derived in the previous subsection, we can efficiently infer the posterior distribution of the connectivity q(S) and the control strength if the link is connected $q(a_{ij}|s_{ij}=1)$. Besides these posterior distributions, we are also interested in the posterior distribution of the latent activity profiles of TFs p(P|E). As they are marginalized out in our model, their posterior distribution can be estimated as:

(19) $$\begin{array}{c} p(P|E)=\int p(P|E,A,S)p(A,S|E)dAdS\\ \approx \int p(P|E,A,S)q(A,S)dAdS \end{array}$$

where we approximate the true posterior p(A,S|E) by the estimated variational posterior q(A,S). According to the model definition, we can derive p(P|E,A,S) as:

(20) $$\begin{array}{c} p(P|E,A,S)=\frac{p(E|P,A,S)p(P)}{p(E|A,S)}\\ =\prod_{t=1}^{M}N\left(p_{t}|\sigma^{2}\Sigma_{p}{\left(A{}^{\circ}S\right)}^{⊤}e_{t},\Sigma_{p}\right) \end{array}$$

where $\Sigma_{p}={\left(\sigma^{-2}{\left(A{}^{\circ}S\right)}^{⊤}(A{}^{\circ}S)+I\right)}^{-1}$. Due to the matrix inversion in Σ_*p*_, the posterior in Equation (19) is not analytically tractable. However, since we only need to infer it once after optimizing the variational posterior q(A,S), we numerically estimate the posterior mean and variance of the activity profiles through Monte Carlo integration.

## 3 Results and discussion

### 3.1 Simulation study

To assess the proposed model, we first generate synthetic data where the true regulatory network, control strengths and activities of TFs are known. To mimic a real network, we take a subset of the connectivity matrix from the real dataset from Iqbal *et al.* (2012) which was obtained from motif analysis. The resulting connectivity matrix contains 353 genes and 20 TFs. The control strength of each interaction and TF activities are sampled from a unit Gaussian. Then 94 gene expression measurements are generated according to the model with noise variance $\sigma^{2}=0.1$. For this validation experiment, we relied on this relatively smaller size network, to be able to run MCMC method long enough to ascertain the convergence and compare the efficiency and accuracy of SITAR against MCMC given the ground truth. As shown in Supplementary Figure S1, not all parameters are converged even after one week‘s run-time.

**Fig. 1:**
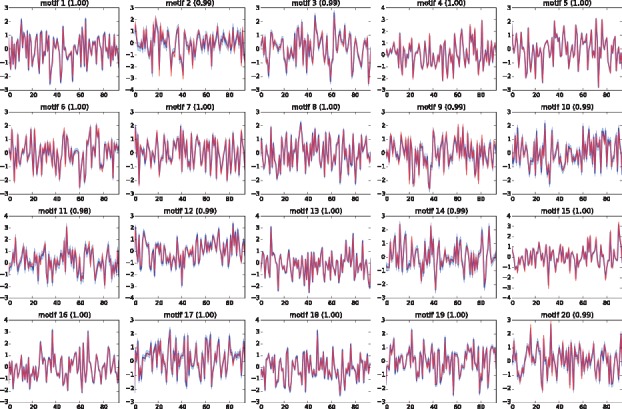
The recovered motif activities (blue) by SITAR are compared with the ground truth activities (red). Their Pearson correlations are shown in the parentheses. For all subplots, x-axis shows the time and y-axis shows the normalized activities of corresponding motifs (Color version of this figure is available at *Bioinformatics* online.)

**Fig. 2:**
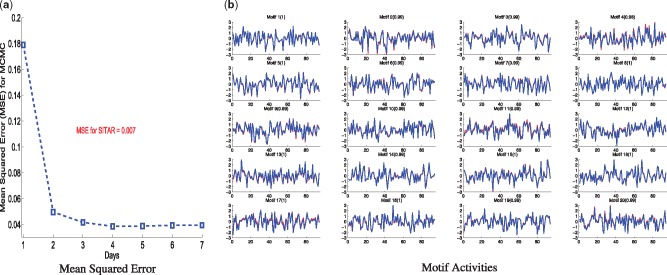
Application of MCMC method (Iqbal *et al.*, 2012) to synthetic data, (**a**) Mean Squared Error (MSE) against the running time for MCMC(single MSE value for SITAR is shown in red), (**b**) Recovered motif activities by MCMC (red) are compared with the ground truth activities (blue), Pearson correlations are shown in the parentheses. For all subplots, *x*-axis shows the time and *y*-axis shows the normalized activities of corresponding motifs (Color version of this figure is available at *Bioinformatics* online.)

We apply the proposed model (SITAR) and the existing MCMC method from Iqbal *et al.* (2012) to the synthetic data. Both methods recover the underlying regulatory network with similar accuracy (93% for SITAR and 92% for MCMC) which is defined as proportion of correctly predicted positive and negative regulatory interactions. TF-gene links were called positive if the corresponding posterior probability was greater than 0.5, negative othewise. The latent motif activities are correctly recovered by both methods, as shown in Figure 2 for MCMC and Figure 1 for SITAR. The control strength and activity profiles are recovered up to an ambiguity of their sign. In order to compare with the ground truth, we correct the sign of the predicted control strength and TF latent activity according to the ground truth control strength. The underlying motivation for this simulation study was to show that given the ground truth network, SITAR is at least as accurate as the MCMC method. At the same time, we want to emphasize on the computational efficiency of our method where one single run of the method took about an hour on a laptop, achieving mean squared error MSE = 0.007 between the input gene expression data and model prediction, which was better than the MCMC even after a week-long run (Fig. 2). Performance of SITAR for synthetic data generated using different noise variances and varying the number of independent gene expression datasets was also studied as shown in Supplementary Tables S1 and S2, respectively.

### 3.2 Application: MTB hypoxia regulatory network

Next, we apply our method to much larger real data from *Mycobacterium tuberculosis* (MTB), involving a large-scale ChIP-seq assay as well as gene expression data measuring response to hypoxia treatment. MTB is known to have a robust hypoxia response network, involving a large number of TFs, facilitating its clinical latency within the host. Earlier analysis of the ChIP-seq data shows that MTB has a highly complex regulatory network, more diversified binding patterns of TFs as compared to previous models of promoter proximal binding, and context-specific occupancy of TF binding sites (Galagan, 2014). For this study, we downloaded 78 samples of gene expression data from GEO (GSE43466, samples GSM1084307 to GSM1084384), which included 10 hypoxia-relevant TF over-expression data, and 68 samples comprising three over-lapping time-series covering hypoxia. We obtained pre-processed ChIP-seq data for 113 MTB TFs (through personal communication with James Galagan, the same data are now publicly available at http://genome.tbdb.org/annotation/genome/tbdb/Resources.html). This constituted a prior topology matrix with 113 columns representing TFs and 3863 rows representing genes with at least one TF connection and for which corresponding expression data was available. The binary entries of this matrix represent the binding of corresponding TF (column) and gene (row). We combined expression data from 78 individial samples in a matrix for all genes present in prior topology matrix, thus obtaining an expression data matrix with 3863 rows representing genes and 78 columns representing samples. This matrix was standardized to zero mean and unit variance.

**Fig. 3:**
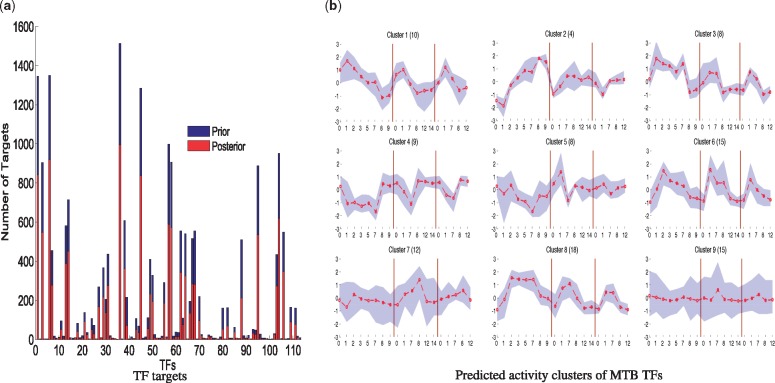
Results of SITAR for MTB data. (**a**) Number of links (targets) for each TF in the prior network based on ChIP-seq data (blue) and posterior links, as predicted significant by SITAR. We used >0.5 cut-off on the posterior probability of links to decide if the link is supported or not. (**b**) Predicted TF activity profiles clustered into 9 clusters (using K-means method). The shaded area represents the activities of cluster members and red line shows the mean profile of the cluster, while the cluster number and number of its members are given in the suplot title. Vertical lines in each plot separate three separate hypoxia experiments (time series SG2, SG6 and SG7 respectively). Out of total 78 samples used in the model, in these plots, we only use 68 samples corresponding to three time-series which are partly replicated, overall cover day0 to day14 of hypoxia, each of them covering a subset of the days, with some overlap with other time series (for detail of the experimental design, see GSE43466). The x-axis shows the time (in Days) for experiments for individual time-series, while y-axis shows the normalized, replicate-averaged, activity of the corresponding TFs

With these many regulators in the model, which is still much less than the total TFs in MTB, a latent factor model with MCMC-based inference would not be feasible while one run of SITAR was completed in just 7 h on a laptop with an 8-core intel processor (E5-2650v2). The results of our methods are shown in Figure 3 where we show that although the prior TF-gene links were obtained from high quality ChIP-seq data (unlike computational motif predictions), there are still large numbers of links for most of the TFs which were switched off by the model after integration with gene expression data (from 21 501 prior interactions obtained form ChIP-seq data, 8645 were switched off by the model). Among the original 113 TFs, there are 14 TFs which were switched off altogether since they have no significant targets. This confirms the quality of prior data on one hand, but also the ability of the model to discard the non-functional links conditional on the gene expression data under use. We also infer the latent activity of all TFs and in Figure 3 we show clusters of activities of TFs showing dynamic patterns in response to hypoxia. Here, we only show the clusters of latent profiles of 99 TFs plotted for three time-series (68 out of the 78 samples used in the model). For all TFs, we also show plots comparing recovered latent activity with gene expression data of corresponding TFs (see Supplementary Figs S3–S9).

**Fig. 4:**
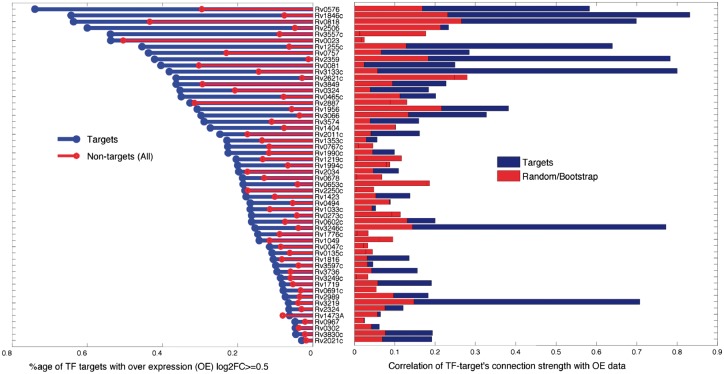
Validation of our predictions against TF over-expression data from Rustad *et al.* (2014). The *y*-axis, showing 54 TFs which have at least 10 significant targets predicted by our method, is shared among two subplots. For each TF, the left panel shows the proportion of targets with log2 fold change (log2FC) ≥0.5 (blue for targets, red for non-targets), while in this data the average ratio of enrichments for targets and non-targets is 3.5. The right panel shows the absolute correlation of over-expression log2FC against the connection strength predicted by the model, blue for targets while red is the background calculated by randomly permuting the connection strength for the given TF targets, averaged over 100 iterations (Color version of this figure is available at *Bioinformatics* online.)

In order to validate our network predictions we used an independent large-scale TF over-expression (TFOE) study in (Rustad *et al.*, 2014; Turkarslan et al., 2015) which was not used to learn the model. This data resulted from a systematic experiment of over-expressing 206 MTB TFs in order to quantify regulatory effects of each transcription factor. We downloaded and extracted the data (http://networks.systemsbiology.net/) for the TFs and genes in our prior network. As shown in Figure 4, we report the enrichment of predicted targets (of given TFs individually) which are showing differential expression in corresponding over-expression experiment ($|\;{\text{log}}_{2}(\text{FC})|\geq 0.5$), for example, for a known regulator involved in hypoxia response (Galagan *et al.*, 2013), RV3133c (DosR), 38% of its 200 significant targets satisfy the over-expression criteria, against 14% among the 3663 non-targets. Although over-expression criteria cannot be considered a completely reliable indicator of TF-gene connection due to complex regulatory control, there are still many regulators, e.g. RV0576, RV1846c, RV2557c among others, whose targets are highly enriched among the differentially expressed genes. The right-hand panel in Figure 4 shows the correlation of the connection strength of TF-gene links predicted by the model against the over-expression indicator, i.e. log2FC. Here again, we have a number of regulators with very strong correlation which is plotted alongside the random background (calculated by randomly reassigning TF-gene connections). Among these, we have RV3133c (DosR) again, with very high correlation, which along with RV0081 are well-known primary hypoxia response regulators, identified in a number of studies in the literature (Galagan *et al.*, 2013; Park *et al.*, 2003). Other known regulators which score highly in this validation analysis include RV3574, RV0324, RV0757 (PhoP), RV1255c and RV2034 among others. Besides these known regulators, we also have few predictions which might be the novel regulators with significant role in hypoxia response and which might be worth analyzing further. These include RV0576 (ArsR family transcriptional regulator), RV1846c (Blal family transcriptional repressor), RV0818 (PhoB) and RV2359 (Zur, zinc uptake regulatory protein) and others as shown in Figure 4.

**Fig. 5:**
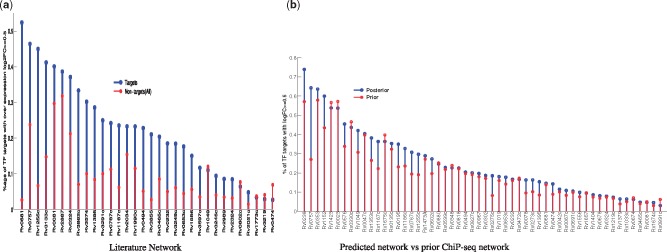
(**a**) Validation of a smaller MTB network published in Galagan *et al.* (2013) against the TF over-expression data. We only used TFs which are in our input network and for which there is OE data. For each TF, we plot the proportion of targets with absolute log2 fold change (log2FC) ≥0.5 (blue for targets, red for non-targets in that network). These analysis was further restricted to TF with at least 10 targets. (**b**) Based on 54 TFs reported in Figure 4, further comparison of our predictions (blue) with the prior network (red) obtained from ChIP-seq data only. The average ratio of enrichments for targets over non-targets for (a) is 3.2 while 1.25 for (b) (Color version of this figure is available at *Bioinformatics* online.)

Furthermore, in order to compare the enrichments shown in Figure 4, we downloaded a relatively smaller hypoxia regulatory network (a Cytoscape session file from Galagan *et al.*, 2013) and performed similar validation analysis using same over-expression data as with our predicted network. After filtering for matching TFs and genes in our network and over-expression data, we have a final network including 39 TFs and 2763 target genes. In Figure 5(a), we show the enrichment of differential targets of TFs in this published network, again plotted alongside non-targets (out of total 2763 genes in that network). Overall, the matching TFs in both networks have similar level of enrichment despite the difference in the number of targets (since our network has a larger number of targets). Also, there are more TFs in our data with highest proportion of differential targets. Lastly, in order to ascertain if our predictions of links were more enriched in differential targets compared to ChIP-seq data (prior), in Figure 5(b), we plot the enrichment for 54 TFs (analyzed earlier in Fig. 4). The majority of predicted TFs are significantly enriched compared to the prior network with only a few exceptions. Overall, this analysis leads us to believe that the model is making biologically meaningful predictions.

## 4 Conclusions

In conclusion, we present a Bayesian sparse factor analysis model coupled with a highly efficient inference scheme to make quantitative inferences of regulatory networks, including binary TF-gene interactions as well as latent activities of TFs, using binding sites and gene expression data in prokaryotic systems. As MCMC is commonly used for inference in these specific type of models, we validate our method against a study with similar modelling scheme for synthetic data where ground truth is known. Our method reproduced the network underlying synthetic data with high accuracy but with much higher efficiency than MCMC-based method. This led us to apply it to much larger real data on *M. tuberculosis* which constitute one of the largest application of regulatory network inference. There is one recent study (Arrieta-Ortiz *et al.*, 2015) where genome-wide network inference was performed on *B. subtilis* data of similar scale, but there are significant differences in the methodology. Inference was done in an iterative two-step procedure, first TF activities were estimated directly from known regulatory interactions using NCA (Liao *et al.*, 2003), which were then used in the prediction of regulatory direction and strength. On the other hand, our approach is significantly different in the sense that we employ a probabilistic model providing simultaneous inference of control strength and latent activities and providing a degree of uncertainty in our estimates.

We perform further validation of our predictions using independent transcriptomics data, compare our predictions against existing network from literature and make novel predictions about the role of certain regulators in MTB’s response to hypoxia treatment. As more and more ChIP-seq and gene expression data becomes available, we believe our method will be a useful tool to make practical inference of large-scale networks regulating gene expression in prokaryotes. Also, since methodology is generic, we can imagine adaptation of our method for other problems in biology and beyond, especially in single-cell RNA-seq applications (see Buettner *et al.*, 2016). Another future direction for our work would be to use a non-linear kernel in our GP formulation or take into account interactions among hidden factors (as in Asif and Sanguinetti, 2011).

## Supplementary Material

Supplementary Data
