# Supplementary material for: Efficient inference for sparse latent variable models of transcriptional regulation
Source: Bioinformatics. 2017 Aug 26;33(23):3776–83. doi: 10.1093/bioinformatics/btx508 (PMC5860323; doi:10.1093/bioinformatics/btx508)
Supplement: Supplementary Data [file btx508_supplementary_materials.pdf]

Supplementary Material: Efficient Inference for Sparse Latent Variable Models of Transcriptional Regulation

Zhenwen Dai*‡1, Mudassar Iqbal†‡2, Neil D. Lawrence ‡3 and Magnus Rattray ‡4

[1,3]Dept. of Computer Science, University of Sheffield, Sheffield, U.K. and Amazon Research, Cambridge, U.K.
[2,4]Faculty of Biology, Medicine, and Health Sciences, University of Manchester, Manchester, U.K.

July 21, 2017

Abstract

In this document, we present two figures, Figure 1 and 2 showing different convergence diagnostics for MCMC method for simulated data as well as two tables showing results for additonal experiments for SITAR using different noise variances used to generate synthetic data (Table 1) and varying the number of indpendent gene expression datasets (Table 2).

Also, for SITAR's application to *Mycobacterium tuberculosis* data, we sought to compare the predicted TF latent activity against their mRNA expression levels (normalized) to ascertain if the inferred activities are indeed very similar or different from the corresponding gene expression data. As we can see from these figures (Figure 3 to 9), many TF latent activities are highly correlated with mRNA expression (perhaps transcriptionally regulated TFs) while others are showing many different behaviours. Please note that in these plots, we have just used hypoxia/aeration time series (SG2, SG6, and SG7). Also, worth noting the fact, there is an inherent sign ambiguity in factor models, hence we have switched the latent profiles where the correlation with corresponding mRNA expression profiles was negative. The shaded area in Figure 3 to Figure 9 represent the standard deviation over replicates and correlation coefficient of latent profiles and observed expression data are given in the title of each plot.

*z.dai@sheffield.ac.uk
†mudassar.iqbal@manchester.ac.uk
‡These two authors contributed equally.

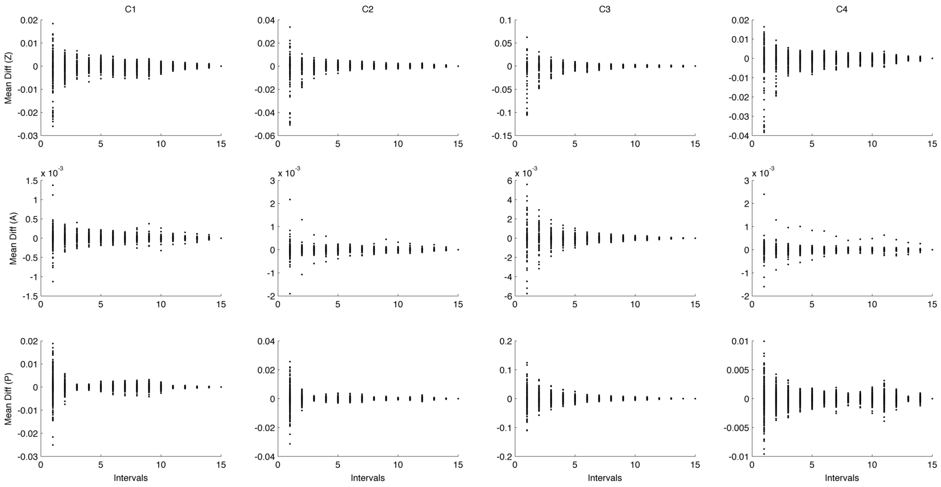

Figure 1: Convergence statistics $\hat{R}$ plotted for 10 equal intervals after discarding 40% of the chains as burn-in

Table 1: Performance, in terms of mean squared error and TF-gene link prediction accuracy, of SITAR for different noise variances used in synthetic data generation.

Noise Variance	MSE	Accuracy
0.001	0.001	97%
0.01	0.0014	96%
0.1	0.007	93%
0.15	0.01	93%

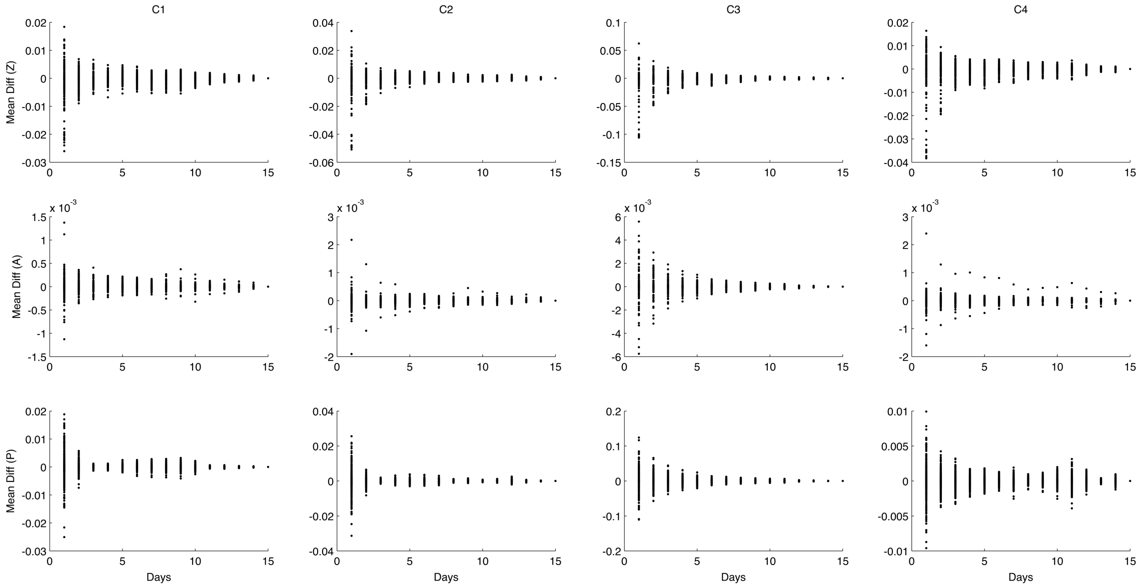

Figure 2: Difference beween posterior mean estimates, for all parameters and all chains, obtained using full-length MCMC chains (one week) and same estimates obtained at different intervals across the length of the chains where each interval is roughly equal to half day.

Table 2: Performance, in terms of mean squared error and TF-gene link prediction accuracy, of SITAR for varying number of indpendent gene expression data.

No. of Eexperiments	MSE	Accuracy
30	0.01	91%
60	0.008	94%
94	0.007	93%

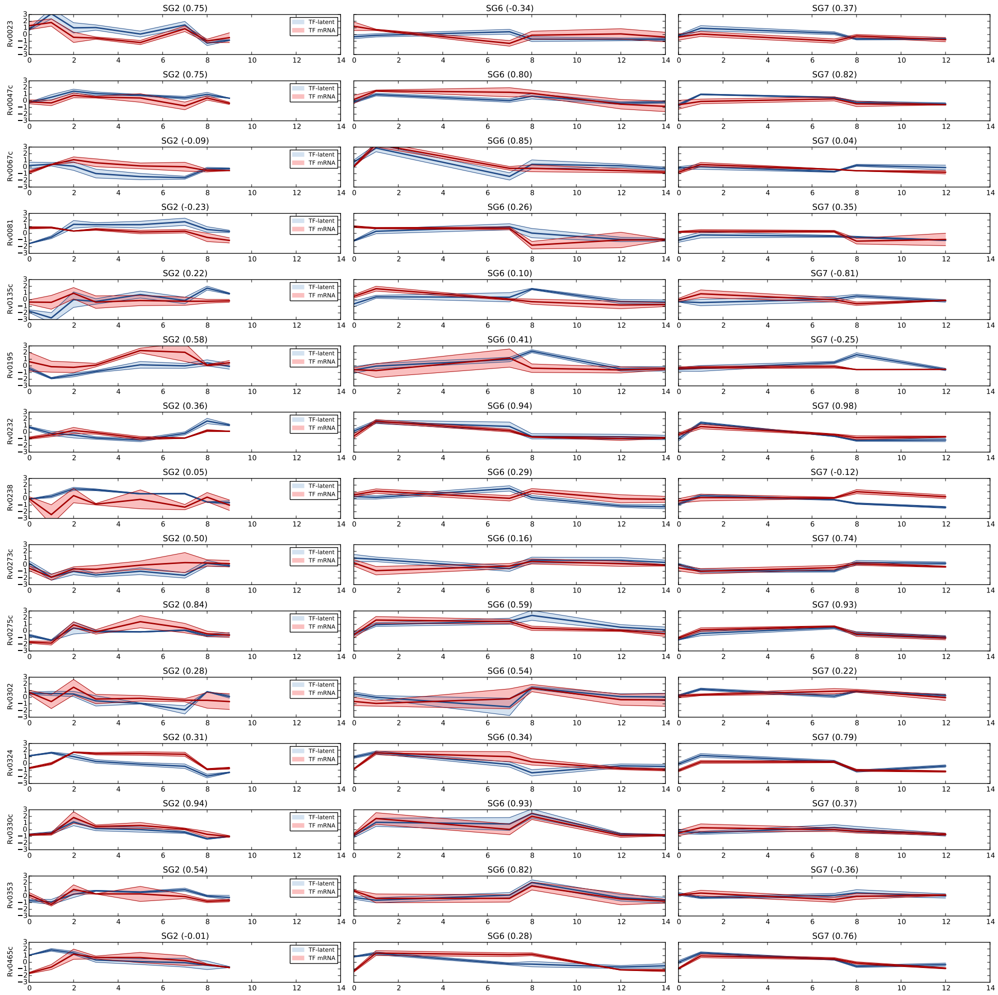

Figure 3: Predicted TF activity vs mRNA expression data.

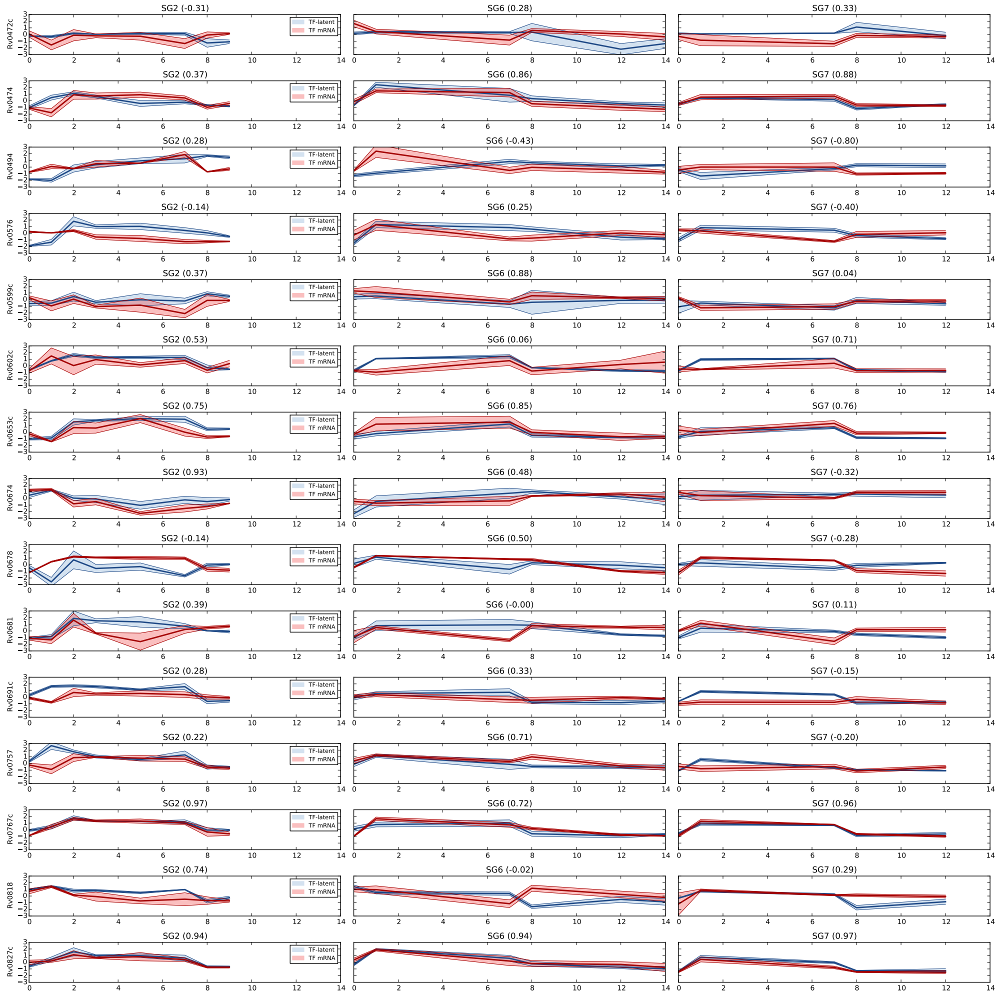

Figure 4: Predicted TF activity vs mRNA expression data

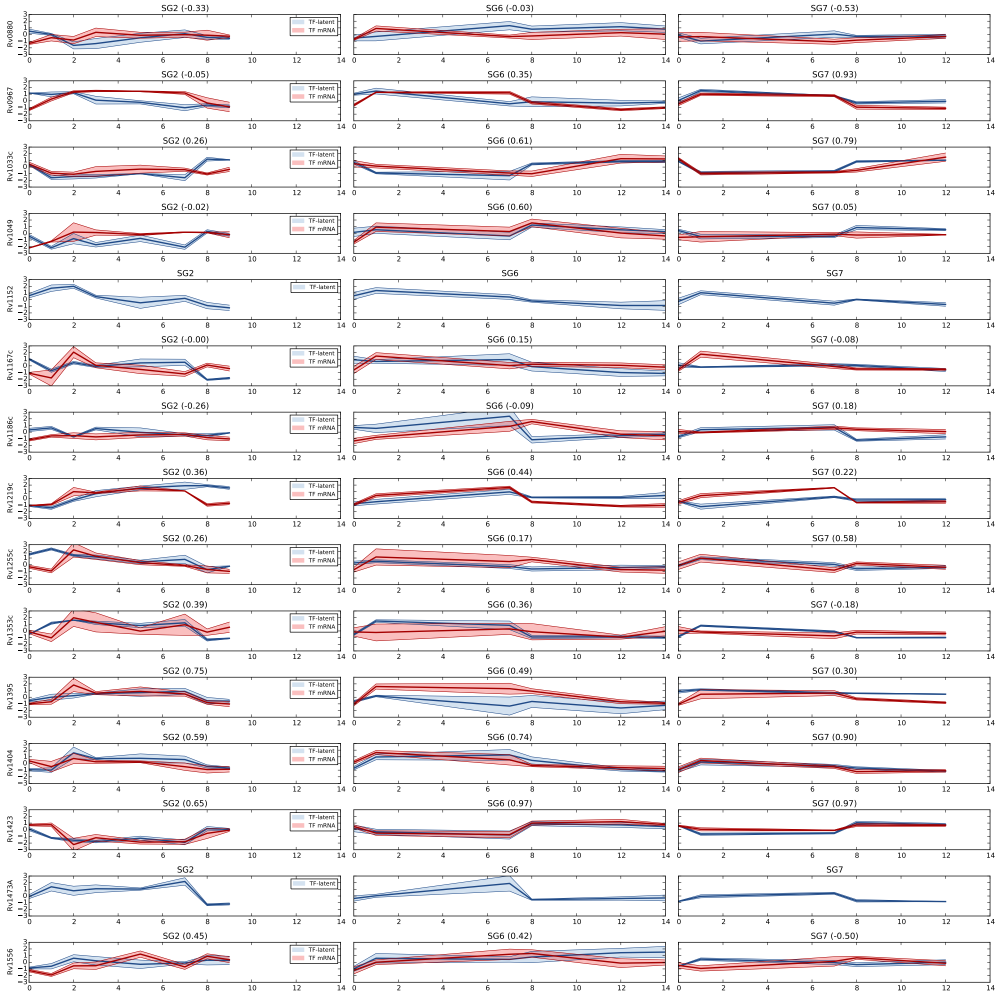

Figure 5: Predicted TF activity vs mRNA expression data

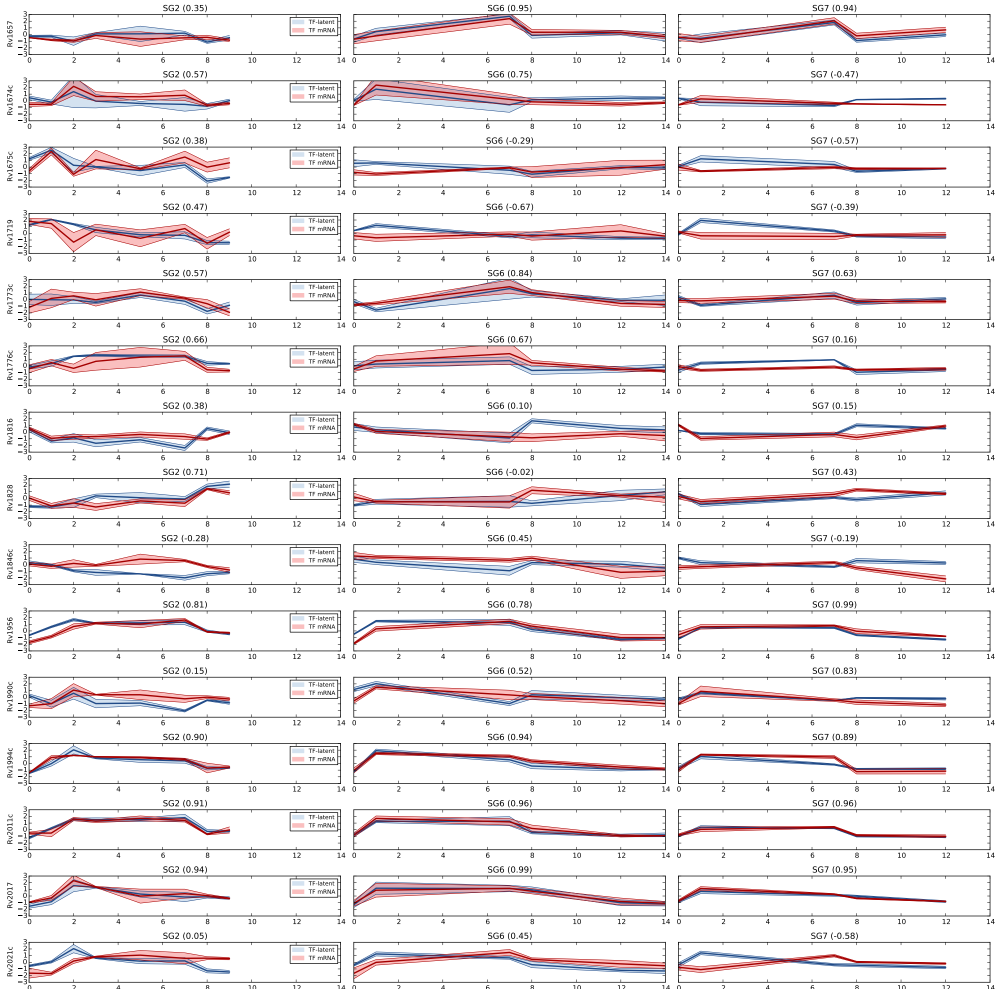

Figure 6: Predicted TF activity vs mRNA expression data

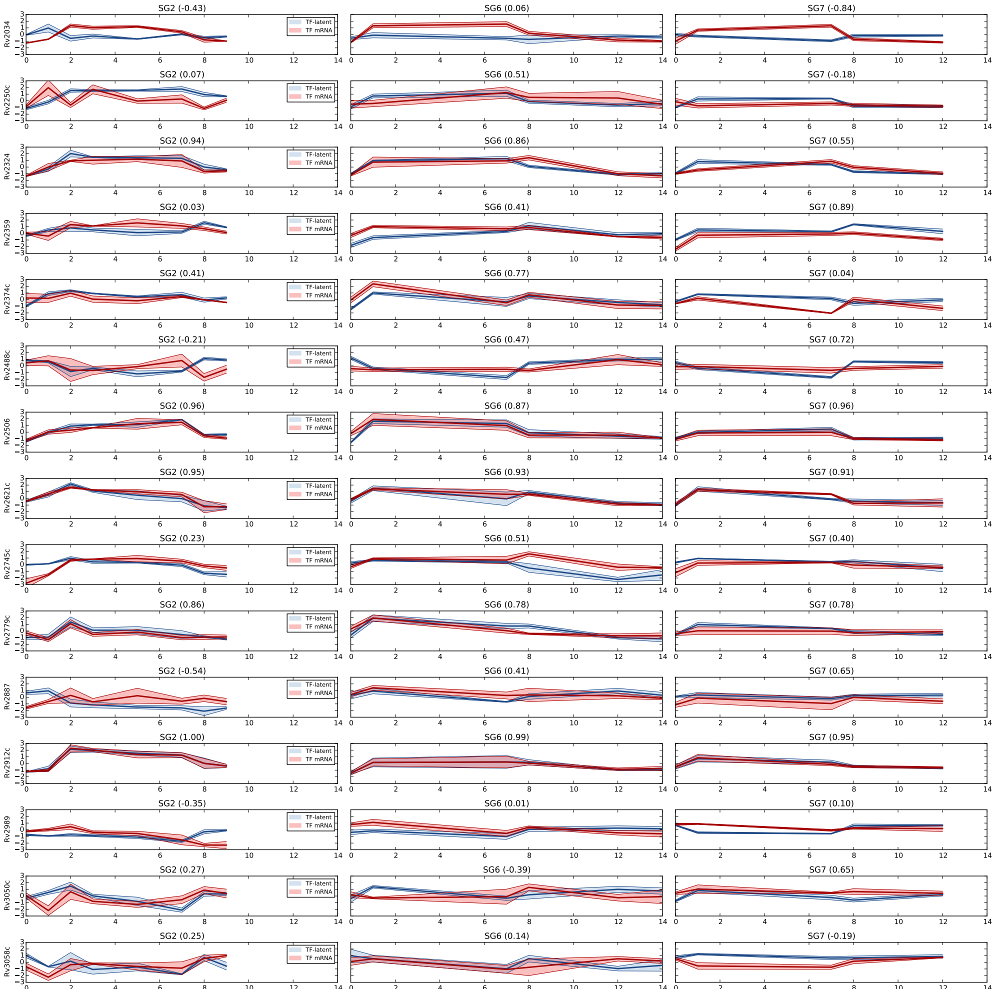

Figure 7: Predicted TF activity vs mRNA expression data

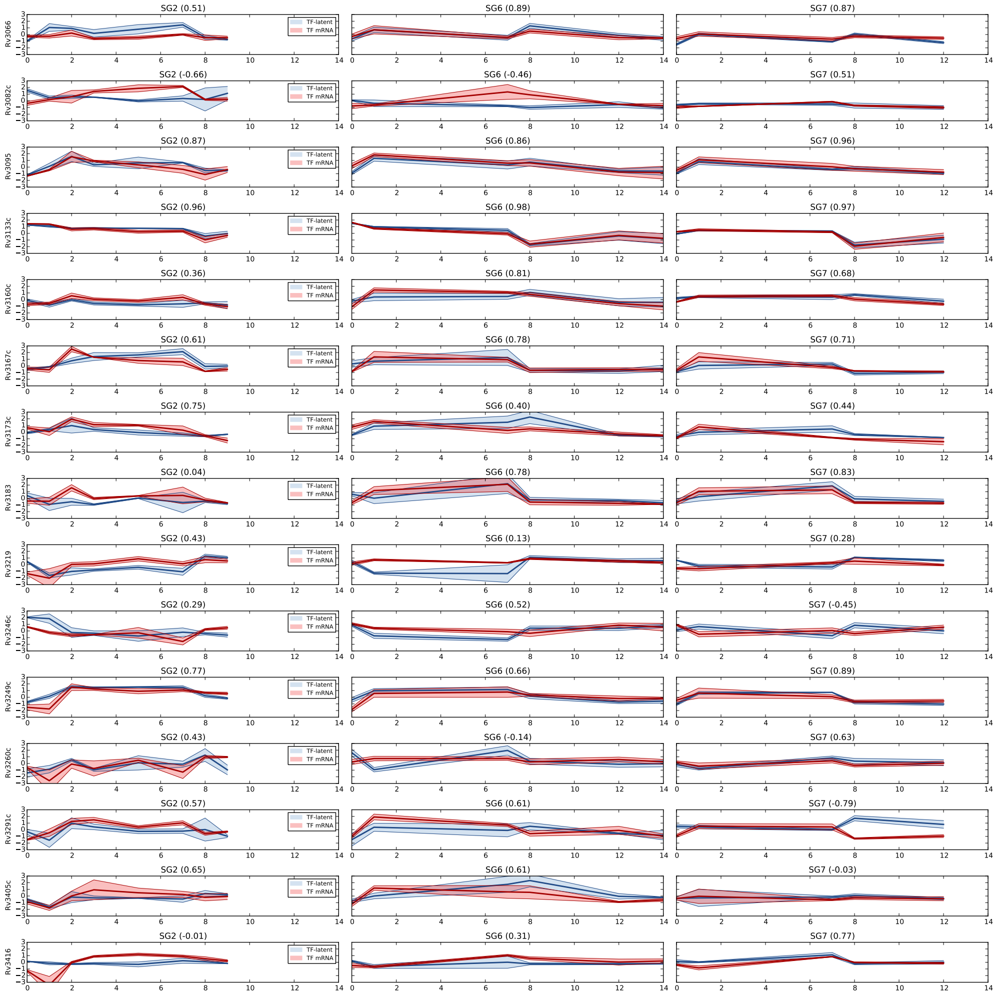

Figure 8: Predicted TF activity vs mRNA expression data

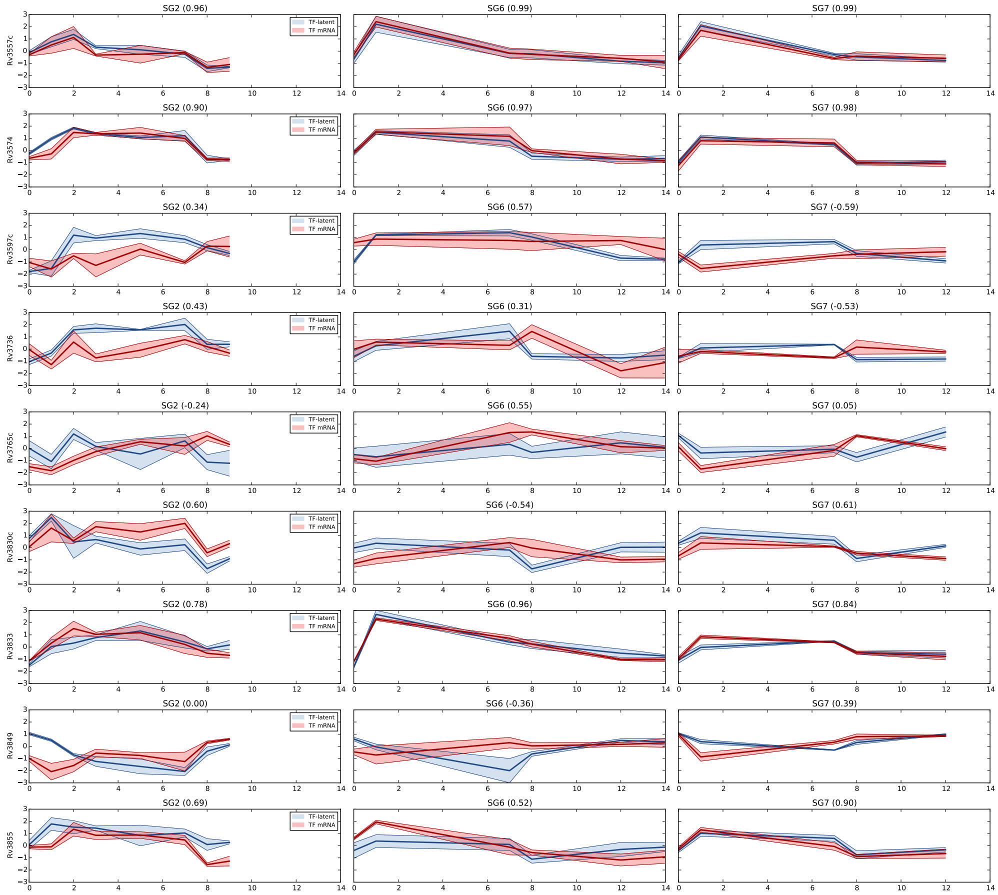

Figure 9: Predicted TF activity vs mRNA expression data
